# Thiamine‐responsive pyruvate dehydrogenase complex deficiency presenting as recurrent muscle weakness: Identification of a novel mutation (p.T111I) in the *PDHA1* gene

**DOI:** 10.1111/ped.15385

**Published:** 2023-01-30

**Authors:** Shunsuke Hayano, Masano Amamoto, Etsuo Naito

**Affiliations:** ^1^ Department of Pediatrics Kitakyushu City Yahata Hospital Fukuoka Japan; ^2^ Department of Pediatrics Japanese Red Cross Tokushima Hinomine Rehabilitation Center Komatsujima Japan

**Keywords:** p.T111I, *PDHA1* gene, pyruvate dehydrogenase complex deficiency, recurrent muscle weakness, thiamine

Pyruvate dehydrogenase complex (PDHC) deficiency is a rare metabolic disorder that affects tissues with high energy demands, such as those of the central nervous system. Pyruvate dehydrogenase complex deficiency is heterogeneous in terms of clinical manifestations. Although the most severe form of PDHC deficiency leads to death in the neonatal period, a mild form involves mild lactic acidosis, mild developmental delay, episodic ataxia, and peripheral neuropathy. In this clinical note, we present a 2‐year‐old Japanese boy diagnosed with thiamine‐responsive PDHC deficiency with a novel mutation in the *PDHA1* gene (p.T111I) accompanied by mild lactic acidosis and repetitive muscle weakness.

The patient was the first child of healthy, unrelated parents and was born at term via normal delivery. His birthweight was 2996 g. His prenatal and postnatal growth and developmental courses were uneventful. The patient was hospitalized at 10 months of age because of acute quadriplegia after an upper respiratory tract infection. He showed marked generalized muscle hypotonia and weakness, ptosis, and diminished deep tendon reflexes.

The plasma lactate level during fasting was 2.18 mmol/L, and the lactate/pyruvate ratio (L/P ratio) was 14.7. Serum creatine kinase and lactate dehydrogenase levels were normal. Brain and cervical spine magnetic resonance imaging (MRI) findings were normal. The cerebrospinal fluid protein level was 19 mg/dl, lactate level was 2.68 mmol/L, and pyruvate level was 0.26 mmol/L without pleocytosis. The level of lactate later dropped to 1.86 mmol/L, and diagnosis could not be confirmed; the patient therefore left the hospital after seven days.

He was hospitalized again at the age of 16 months because of recurrent acute quadriplegia. Plasma lactate levels were 3.6–4.8 mmol/L, pyruvate levels were 0.28–0.46 mmol/L, with a normal L/P ratio. The plasma alanine level was 623.8 μmol/L (normal 361 ± 88), with a high alanine/lysine ratio (3.75). Computed tomography (CT), single photon emission CT, MRI, and magnetic resonance spectroscopy of the brain were normal. From these data, we suspected a PDHC deficiency, and the child received adjuvant therapy with 100 mg/day of thiamine. He was discharged from the hospital after 3 weeks.

The DNA sequence of the patient's X‐linked E1a subunit revealed a novel p.T111I mutation in exon 4 (Figure 1). Although we did not assay the PDHC activity, we diagnosed a PDHC deficiency because of his clinical manifestations and the mutation of the *PDHA1* gene. With reference to a report by Naito et al.,^1^ the patient continued oral thiamine treatment even after symptom improvement.

**FIGURE 1:**
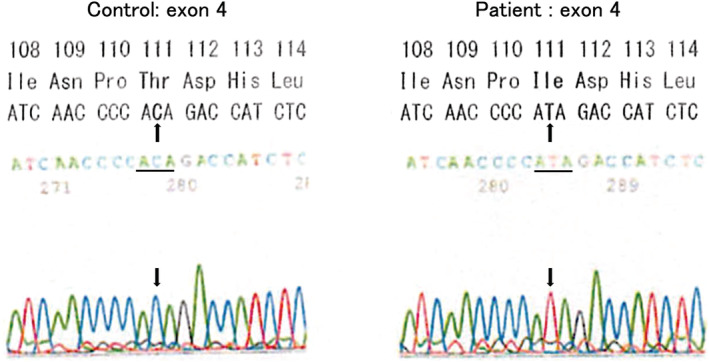
Sequence analysis of exon 4 of the patient's *PDHA1* gene allowed the identification of a C→T transition corresponding to the p.T111I heterozygous mutation

At 24 months old, after a fever of exanthema subitum, the patient was hospitalized because of inability to walk and sit, marked ptosis, inactivity, diminished deep tendon reflexes, and irritability. Based on his clinical symptoms and elevated plasma lactate and pyruvate levels, we diagnosed severe neurological deterioration due to PDHC deficiency. He had been taking thiamine, and we prescribed a higher dose of thiamine (500 mg/day). For 2 days, neurological symptoms and lactate and pyruvate levels did not improve; we therefore increased the dose to 1,000 mg/day. Thereafter, neurological symptoms improved, so we reduced the dose to 750 mg/day and then to 500 mg/day.

After discharge, he continued taking thiamine at 500 mg/day, and 8 weeks after the third attack, he reached normal exercise levels and deep tendon reflexes, his lactate and pyruvate levels decreased. His activity resumed as usual, growth and development are proceeding well, with no further attacks for 18 months.

In this patient, the acute clinical symptoms and long‐term management seemed to be improve by high‐dose thiamine therapy. Certain mutations in exon 3 outside the thiamine pyrophosphate (TPP)‐binding site, such as H44R, V71A, R88S, and G89S, and in exon 7 within the TPP binding site, such as F205L and L216F, are associated with a good response to thiamine treatment.^2^, ^3^ Another case of a p.C101F mutation in exon 4 (near p.T111I) has been reported as thiamine responsive – he showed low PDHC activity in the presence of low TPP, but his PDHC activity significantly increased at a high TPP concentration. It is likely that PDHC activity also increased in this case at a high TPP concentration.

This disease must be suspected in recurrent flaccid limb paralysis. Measurement of PDHC activity and genetic analysis are useful for diagnosis, but lactate and pyruvate levels as well as clinical findings are also important for diagnosis. Our patient's PDHC deficiency was caused by a p.T111I mutation that seems to be thiamine responsive from his clinical course. However, the thiamine supplementation dosage to treat acute disease and prevent recurrent attacks must be clarified in future studies.

We received ethical committee approval in our hospital and informed consent from the patient's parents.

## AUTHOR CONTRIBUTIONS

S.H. wrote the manuscript. S.H. and M.A. were responsible for patient diagnosis and treatment. E.N. assisted in the preparation of the manuscript. All authors read and approved the final article.

## CONFLICT OF INTEREST

The authors declare no conflict of interest.
